# Homeostatic alterations related to total antioxidant capacity, elemental concentrations and isotopic compositions in aqueous humor of glaucoma patients

**DOI:** 10.1007/s00216-021-03467-5

**Published:** 2021-06-25

**Authors:** Marta Aranaz, Marta Costas-Rodríguez, Lara Lobo, Montserrat García, Héctor González-Iglesias, Rosario Pereiro, Frank Vanhaecke

**Affiliations:** 1 Department of Physical and Analytical Chemistry, University of Oviedo, Avda. Julián Clavería 8, 33006 Oviedo, Spain; 2 Department of Chemistry, Atomic & Mass Spectrometry – A&MS Research Unit, Ghent University, Campus Sterre, Krijgslaan 281 - S12, 9000 Ghent, Belgium; 3 Instituto Oftalmológico Fernández-Vega, Avda. Fernández-Vega 34, 33012 Oviedo, Spain; 4 Instituto Universitario Fernández-Vega, Fundación de Investigación Oftalmológica, Universidad de Oviedo, Avda. Fernández-Vega 34, 33012 Oviedo, Spain

**Keywords:** Elemental concentration, Isotopic composition, Inductively coupled plasma–mass spectrometry, Aqueous humor, Pseudoexfoliation glaucoma

## Abstract

Glaucoma is a multifactorial eye disease, characterized by progressive optic neurodegeneration. Elevation of the intraocular pressure is the main risk factor for glaucoma and is a consequence of an imbalance in the aqueous humor hydrodynamics, the physiology of which is influenced by the homeostatic equilibrium of essential elements, oxidative stress, and antioxidants. The aim of this work was to study local alterations in glaucomatous patients from two different, but connected, points of view: (i) the total antioxidant capacity (as an indicator of oxidative damage) and (ii) the concentration of mineral elements and their isotopic composition. Such objective was pursued using aqueous humor from patients diagnosed with pseudoexfoliation glaucoma (PEXG, n = 17) and primary open-angle glaucoma (POAG, n = 5) and age-matched control subjects (n = 16). The total antioxidant capacity (TAC) was examined in both aqueous humor and 60 serum samples (n = 20 controls, n = 20 for PEXG, and n = 20 for POAG), both showing higher TAC for the glaucoma population. The concentrations of the essential mineral elements (Cu, Fe, Mg, Na, P, and Zn) and the isotopic compositions of Cu and Zn were determined in aqueous humor using single-collector and multi-collector inductively coupled plasma–mass spectrometry, respectively. Significant differences were established for Mg and P levels when comparing the results for glaucomatous patients with those for the control population (p < 0.01 and p < 0.05 for Mg and P respectively, ANOVA and Kruskal-Wallis). The Zn isotopic composition was significantly shifted from that for the control population for PEXG patients. A significant difference in the isotopic composition of Zn was also established between the PEXG and POAG glaucoma cohorts.

**Graphical abstract:**

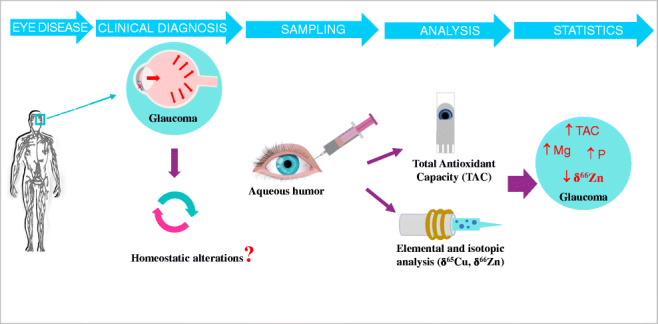

**Supplementary Information:**

The online version contains supplementary material available at 10.1007/s00216-021-03467-5.

## Introduction

Glaucoma comprises a complex group of neurodegenerative disorders, affecting nearly 80 million people worldwide, and is considered one of the leading causes of irreversible blindness [1]. This multifactorial disease is characterized by optic nerve damage and retinal ganglion cell (RGC) death, which leads to visual field loss. During the initial stages of the disease, glaucoma might evolve unnoticed, and at first clinical diagnosis, loss of RGCs can amount up to 40% already [2]. While its etiology remains so far unknown and its incidence increases with age, elevation of intraocular pressure (IOP) is the main risk factor for the development of glaucoma [1, 3]. The most prevalent subtypes of glaucoma include primary open-angle glaucoma (POAG) and pseudoexfoliation glaucoma (PEXG), sharing a dysfunction of the aqueous humor dynamics that results in elevated IOP. While POAG is usually associated with an alteration in the cell physiology of the trabecular meshwork [3], PEXG is characterized by the progressive accumulation of abnormal fibrillar protein aggregates in the anterior chamber of the eye [4].

Aqueous humor is a colorless intraocular fluid, vital in the physiology of the eye, that is produced by the ciliary body. It derives from plasma, within the capillary network of the ciliary processes, being secreted at a rate of 2–2.5 μL/min in the ocular anterior chamber. Once secreted, aqueous humor is drained mainly via the trabecular meshwork and, to a lesser extent, the suprachoroidal space. Circulating aqueous humor contributes to maintaining proper IOP and structural integrity of the globe; provides substrates and removes metabolites from the cornea, lens, and trabecular meshwork; and participates in immune and antioxidant responses [5]. Oxidative stress contributes to the development and progression of glaucoma [6]. Although under physiological conditions, oxidative stress can be counteracted by the eye antioxidant defense system, relying on a vast variety of compounds, including superoxide dismutase (SOD), glutathione peroxidase (GPX), catalase (CAT), and metallothioneins (MTs), the pathophysiological alterations during glaucoma onset and progression may affect the aqueous humor dynamics, triggering oxidative damages in the trabecular meshwork, which results in IOP elevation and RGC death [7, 8]. Therefore, the determination of the aqueous humor antioxidant capacity may reflect the ability to scavenge free radicals, thus providing information on potential imbalance of oxidative species and antioxidants.

It is well known that essential mineral elements participate in numerous biological processes, including antioxidant response, and their determination in biological fluids provides information on the health status of individuals [9–11]. For instance, trace elements such as Fe, Cu, Se, and Zn are required as cofactors for the activity of a wide range of antioxidant enzymes [12]. Most publications focusing on essential mineral element determination deal with analysis of whole blood or serum. Access to aqueous humor is limited, both in terms of availability (typically 100–200 μL for aqueous humor) and invasiveness, such that literature on the topic is scarce. Nevertheless, association between changes in aqueous humor elemental concentrations and the occurrence of several eye diseases has been reported recently. Particularly, higher Co, Fe, Zn, and Cd levels have been found in the aqueous humor of dry age-related macular degeneration (AMD) patients compared to a control population, whereas Cu levels were significantly reduced in the AMD group [13]. In contrast, higher levels of Cu have been reported in patients with cytomegalovirus retinitis [14]. Additionally, significant differences have been found for both Zn and Fe levels between glaucoma patients and control subjects, supporting the hypothesis that in this ocular fluid, deviating trace element concentrations might signal problems and may thus also provide a more profound insight into the pathogenesis of neurodegenerative eye diseases [15]. Changes in their isotopic composition may also provide relevant information, different from that embedded in the element concentrations [16]. However, to the best of the authors’ knowledge, isotopic compositions have not been evaluated in aqueous humor yet. High-precision isotopic analysis by multi-collector (MC) inductively coupled plasma–mass spectrometry (ICP-MS) of samples with low absolute quantities of analyte is still a challenge, as this approach typically requires analyte amounts between 200 and 500 ng depending on the target element and the instrument settings used (e.g., sample introduction system, interface, and resolution mode). Strategies aiming at reducing the amount of sample (and analyte) required for isotopic analysis are of interest, in particular within a clinical context [17–19].

In this vein, the goal of the present work was to investigate potential alterations in the aqueous humor of glaucomatous patients, compared to control subjects, in terms of their antioxidant capacity, elemental concentrations, and isotopic compositions. It should be noted that it was rather challenging from the analytical point of view to reach such objective due to the limited amount of sample available (most of the samples had a volume of 50–150 μL). The antioxidant capacity was evaluated to study whether the oxidative status is altered as a result of the disease. Multi-elemental analysis (Cu, Fe, Mg, Na, P, Zn) and, for the first time, also isotopic analysis (Cu and Zn) were accomplished using ICP-MS and MC-ICP-MS respectively, with the aim of revealing potential homeostatic alterations for mineral elements related to glaucoma.

## Experimental

### Selection of individuals

All participants were recruited at the Institute of Ophthalmology Fernández-Vega (Oviedo, Spain), signed an informed consent, and underwent complete ophthalmologic examination, including slit-lamp biomicroscopy and fundoscopy in both eyes. In addition, glaucomatous patients underwent specific ophthalmic tests (gonioscopy, perimetric field loss, IOP, analysis of nervous fibers). The diagnostic criteria for POAG and PEXG were the presence of characteristic optic-disc damage (e.g., vertical cup-to-disc ratio > 0.3, thin or notched neuroretinal rim, or disc hemorrhage) with the corresponding characteristic changes in the visual field and the presence of an open anterior chamber angle (Shaffer grade III or IV). POAG patients did not present secondary causes of optic neuropathy, while the subjects with PEXG exhibited deposits of exfoliative material on the anterior lens surface and/or iris in one or both eyes, as revealed during slit-lamp examination. No subjects involved in this study presented any other relevant ocular pathology, such as retinopathies or maculopathies.

A total of 38 participants were recruited for collection of aqueous humor: 17 subjects diagnosed with PEXG, 5 subjects suffering from POAG, and 16 age-matched controls, all suffering from cataract. With the aim of avoiding misclassification, a detailed clinical history was obtained from all participants. Gender, average age, and age range, together with comorbidities, are collected in the Supplementary Information (ESM Table S1) for each group. Aqueous humor samples were collected at the beginning of cataract surgery: a 27-gauge needle was placed in the anterior chamber and the aqueous humor was removed, while taking care to avoid sample contamination. Samples were immediately frozen and stored at −80 °C until analysis.

In addition, blood samples were collected from newly recruited glaucoma patients (20 PEXG and 20 POAG) and 20 control subjects, whose demographic conditions are summarized in the ESM (Table S2), to validate the analysis of antioxidant capacity. Blood was collected in 5-mL Z Serum Sep Clot Activator tubes coated with microscopic silica particles, which activate the coagulation process (Vacuette, Madrid, Spain). Tubes were centrifuged at 1800*g* for 18 min at 4 °C and the supernatant (serum) was stored at −80 °C until use.

### Assessment of total antioxidant capacity

The capability to counteract oxidative stress-induced damage in cells can be determined by measuring the total antioxidant capacity (TAC). Here, it was measured by means of an e-BQC lab device (BioQuoChem, Spain). The e-BQC lab system is based on the measurement of a redox potential, directly providing the antioxidant capacity as the charge/period (μC). Apart from the total antioxidant capacity, the e-BQC lab system also allows distinguishing between fast- and slow-acting antioxidants, while the TAC corresponds to the sum of fast and slow antioxidants. Measurements were carried out with 30 μL of sample, recovered after the measurement, permitting the subsequent multi-elemental and isotopic analysis. Due to the low amount of aqueous humor available, this method was selected as the best option. In addition, a quantitative colorimetric assay (Total Antioxidant Capacity Assay Kit, Sigma-Aldrich) was used for further validation of the methodology in serum samples from newly recruited glaucoma patients. The latter kit expresses the TAC in Trolox® equivalents.

### Sample preparation for elemental and isotopic analysis

Aqueous humor samples with a volume between 10 and 450 μL were available for this work. Samples were first acid digested at 110 °C overnight using 500 μL of 14 M HNO_3_ (Trace Metal Grade; Fisher Scientific, UK) and 125 μL of 9.8 M H_2_O_2_ (Sigma-Aldrich, Spain). This mixture was then evaporated until dryness at 90 °C and reconstituted in 8 M HCl and 0.001% H_2_O_2_. The digest thus obtained was subjected to chromatographic isolation using AGMP1 resin (Biorad, Spain), as described elsewhere [20, 21]. Optima grade HCl (12 M) was acquired from VWR (VWR International, Barcelona, Spain). The isolation procedure allows to successively isolate Fe, Cu, and Zn by eluting first the matrix fraction in 8 M HCl + 0.001% H_2_O_2_, then Cu in 5 M HCl + 0.001% H_2_O_2_, followed by Fe and Zn in 0.6 M HCl and 0.7 M HNO_3_, respectively. After the chromatographic separation, the remaining matrix fraction was evaporated until dryness at 90 °C and the residue subsequently reconstituted in 500 μL of 0.28 M HNO_3_ for P, Mg, and Na determination. The same procedure was carried out for the Fe fraction. The pure Zn fraction was evaporated until dryness (at 90 °C) and taken up in 200 μL of 14 M HNO_3_. This was carried out twice to assure total removal of Cl^−^. The final residue obtained upon drying was then reconstituted in 500 μL of 0.28 M HNO_3_. A small aliquot of this solution was used for Zn elemental determination and the rest of the fraction was subjected to isotopic analysis. The pure Cu fraction was evaporated until dryness (at 90 °C) and the residue taken up in 8 M HCl and 0.001% H_2_O_2_ and subjected to a second isolation to assure complete removal of Na [22]. The purpose of this procedure is to avoid ^40^Ar^23^Na^+^ spectral interference affecting the ^63^Cu^+^ ion signal. The pure Cu fraction thus obtained was dried and the residue reconstituted in 200 μL of 14 M HNO_3_. This was carried out twice. The final residue obtained upon drying was then reconstituted in 500 μL of 0.28 M HNO_3_ for Cu determination and isotope ratio measurement.

### Instrumentation

Elemental analysis was accomplished using an Element 2 and Element XR single-collector sector-field ICP-MS instruments (Thermo Scientific, Germany). The trace elements (Fe, Cu, Zn) were measured at Ghent University (Belgium), whereas the minor elements Na, Mg, and P were determined at the University of Oviedo (Spain). Quantification was accomplished via external calibration for which commercially available single-element standards (1000 mg L^−1^ stock solutions for Fe, Cu, Zn, P, Mg and 10,000 mg L^−1^ stock solution for Na) from Merck (Germany) were used. Ga (Merck, Germany) was used as an internal standard, correcting for potential signal instability and matrix effects. All dilutions were made in 0.28 M HNO_3_. The nuclides ^23^Na, ^31^P, ^24^Mg, ^63^Cu, ^56^Fe, and ^66^Zn were monitored at medium mass resolution (R = 4000).

Isotopic analysis was performed using a Neptune XT MC-ICP-MS instrument (Thermo Scientific, Germany), equipped with a high-transmission jet interface (Jet-type Ni sampling cone and X-type Ni skimmer cone) and an aerosol desolvation system (Aridus II) for sample introduction into the plasma, in operation at Ghent University. Cu and Zn isotope ratio measurements were carried out at medium (pseudo) mass resolution at a concentration level of 5 and 20 μg L^−1^, respectively. Correction for mass discrimination was performed by means of a combination of internal normalization (solutions doped with Ga for Cu isotope ratio measurement and with Cu for Zn isotope ratio measurement) and external correction using a standard measured in sample-standard bracketing sequence. The isotopic certified reference materials NIST SRM 976 (National Institute of Standards and Technology, MD, USA) and IRMM-3702 (Institute of Reference Materials and Measurements, Belgium) were used as external standards for Cu and Zn isotope ratio measurements, respectively. In addition, in-house standards, previously characterized for their isotopic composition [21, 23], were measured to assure the quality of the isotope ratio measurements.

### Statistical analysis

All data were analysed with the SPSS software version 25.0 (IBM Corp., Armonk, NY, USA). Statistical analysis between groups relied on the chi-square or Fisher’s test for categorical variables, whereas continuous variables were compared with the Mann-Whitney U test. Normality of the data distribution was evaluated with the Shapiro-Wilk test on elemental, isotope ratio, and antioxidant capacity data. One-way analysis of variance (ANOVA) (with Bonferroni’s post hoc test) or the Kruskal-Wallis test (with Dunn’s post hoc test) was used to evaluate differences between the three groups (control, PEXG, and POAG), depending on the normal distribution. In all cases, significance was established at p < 0.05.

## Results and discussion

### Study subjects

Demographic characteristics of the recruited 17 PEXG and 5 POAG patients and 16 control individuals for aqueous humor analysis are compiled in ESM Table S1. There was no significant difference (*p* > 0.05) in terms of age, gender, and risk factors when comparing the glaucomatous patients with the controls. An additional logistic regression analysis was carried out to determine the effects of these features on the prediction of glaucoma and to check for potential confounders, observing no significant effects. ESM Table S2 summarizes the demographic characteristics of the newly recruited glaucoma patients (20 PEXG and 20 POAG) and 20 controls used for the confirmation of the determination of the antioxidant capacity. No statistically significant differences were obtained (p > 0.05) in terms of age, gender, and risk factors when comparing glaucomatous patients with controls, and none of the characteristics is a potential confounder in the prediction of glaucoma.

It must be highlighted that POAG patients were included in this study, despite the low number of aqueous humor samples available, in an attempt to identify possible changes among the most prevalent types of glaucoma, which are clinically and genetically distinct. POAG is primarily the result of a complex pattern of heritage, whereas PEXG is a secondary form of glaucoma in many patients with exfoliation syndrome. However, the similar antioxidant capacity obtained for both glaucomatous groups implies upregulation of the antioxidant systems in both cases.

### Antioxidant capacity of aqueous humor and serum

**Fig. 1:**
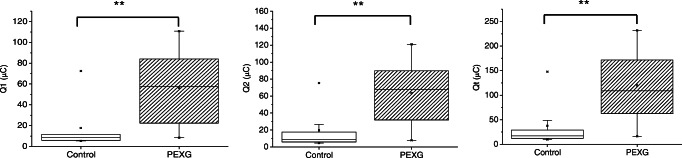
Box plots for Q1, Q2, and Qt (μC) measured in aqueous humor of patients suffering from pseudoexfoliation glaucoma (n = 14) and of controls (n = 12). n.s., not significant; *p-value <0.05; **p-value <0.01; ***p-value <0.001

The antioxidant capacity was measured in 16 aqueous humor samples from glaucomatous patients (14 PEXG and 2 POAG) and 12 controls, selected from all the subjects considering the limitation of low sample availability. The results, expressed as Q1 (fast antioxidants), Q2 (weak antioxidants), and Qt (TAC), are shown in Fig. 1. Statistical analysis provided significant differences between controls and PEXG for Q1 (p = 0.005, Dunn post hoc test), Q2 (p = 0.003, Dunn post hoc test), and Qt (p = 0.003, Dunn post hoc test). As can be noted from the box plots in Fig. 1, Q1, Q2, and Qt were higher in PEXG as compared to the control group, and therefore, the glaucoma patients exhibited higher antioxidant capacity than the control subjects. Age-related variations cannot be the cause of the differences in antioxidant capacity observed since both (glaucoma and control) groups were age-matched. The antioxidant capacity obtained for POAG patients (Q1 = 55 ± 15 μC; Q2 = 57 ± 11 μC; Qt = 112 ± 26 μC; n = 2) falls within the range of values for the PEXG subjects. Due to the low number of samples available for analysis, the antioxidant capacity for the POAG patients is not plotted in Fig. 1.

Our results, showing a significantly higher TAC for glaucoma patients than for the control group, do not seem to agree with previous studies reported in literature for several neurodegenerative ocular disorders, including glaucoma. Ferreira et al. [24] observed that the aqueous humor of patients diagnosed with PEXG and POAG presented lower total reactive antioxidant potential (equivalent to antioxidant status) than subjects with cataract. Conversely, Ergan et al. [25] showed a higher aqueous humor total antioxidant status in patients with glaucoma (POAG and PEXG) than in the control group (cataract). According to Beyazyildiz et al. [26], the TAC of the aqueous humor in patients presenting exfoliation syndrome were similar to the control subjects, without significant difference. In a Spanish population, Zanon-Moreno et al. [27] found a lower antioxidant activity in the aqueous humor of POAG patients when compared to controls, using a colorimetric assay for indirectly determining antioxidants. Regarding other eye diseases, Sawada et al. [28] observed that significant increases in the levels of antioxidant activity (specifically SOD) correlated with the severity of cataract. Another publication reported that patients with retinitis pigmentosa (RP) also showed a lower total antioxidant capacity as compared to the control population [29]. These authors also showed reduced SOD3 activity for RP patients. This extracellular SOD regulates concentrations of reactive oxygen and nitrogen species and, therefore, a decreased level may be responsible for the inflammatory process occurring in this retinal degeneration.

Considering the conflicting results published to date and with the aim of further confirming the validity of our analysis in aqueous humor and figuring out whether the TAC reflects the situation at the systemic level, the antioxidant capacity was measured in 60 serum samples from newly recruited patients with PEXG (n = 20), POAG (n = 20), and control subjects (n = 20), by means of both the e-BQC system and the Total Antioxidant Capacity Assay Kit (which is more widely used for TAC experiments). The results obtained are summarized in Fig. S1 (see ESM). Both methodologies for TAC analysis provided higher values for glaucomatous patients (PEXG and POAG) as compared to controls. Specifically, the Total Antioxidant Capacity Assay Kit (panel A, in nmol Trolox levels) provided higher antioxidant status in PEXG and POAG patients when compared to control subjects with statistically significant differences (p < 0.001 and p = 0.012, Dunn post hoc test, respectively), while no differences were found when comparing POAG and PEXG cases (*p* = 0.549, Dunn post hoc test). Similarly, the results obtained using the e-BQC device (panels B to D) showed higher antioxidant capacity in glaucoma patients when compared to control subjects, with significant differences between (i) controls and PEXG (p < 0.001 for Qt; p < 0.001 for Q1; p = 0.003 for Q2) and (ii) POAG and PEXG (p = 0.007 for Qt; p < 0.001 for Q1; p = 0.147 for Q2). No significant differences were found between controls and POAG patients (p = 0.089 for Q1, p = 0.523 for Q2, and p = 0.423 for Qt). Overall, in this study, higher antioxidant capacity has been found for both aqueous humor and serum in individuals diagnosed with glaucoma as compared to the control cohort.

Oxidative stress markers have been evaluated in glaucoma patients at local and systemic levels. For instance, malondialdehyde (MDA), a marker of peroxidative damage, was found to be significantly increased in serum and aqueous humor samples from POAG patients [7]. Another publication reported higher levels of MDA and lipofuscin, a marker linked with age-related disorders, in serum samples from glaucomatous patients as compared with controls, all having cataract. Moreover, the total SOD activity was significantly lowered in the case of glaucoma group [8]. This antioxidant enzyme, which catalyses the reaction of superoxide anion molecules, leading to O_2_ and H_2_O_2_ formation, plays an important role in the metabolism of oxygen free radicals. Therefore, the reduced aqueous humor levels of SOD found in glaucomatous patients (POAG) could lead to trabecular meshwork disruption [30]. In accordance with those results, another publication reported lower expression of SOD1 (the cytosolic copper- and zinc-containing SOD) in patients with POAG in peripheral blood samples in comparison with the control group. Nevertheless, GPX1 and SOD2 (the mitochondrial manganese-containing SOD) gene expression were higher in POAG patients than in control subjects [24, 31]. Both enzymes play a key role in the antioxidant system by means of superoxide by-products conversion and hydrogen peroxide removal, respectively. An increase in SOD activity was also observed in aqueous humor from glaucomatous patients compared to a cataract group [32]. Finally, studies carried out in serum of glaucoma patients reported comparable TAC levels as in cataract subjects used as controls [8].

Considering that the antioxidant capacity is inversely proportional to oxidative stress, the aqueous humor of patients with glaucoma seems to present either lower levels of free radicals or higher levels of antioxidants, which is opposite to what one would expect during the development of such disease [33]. Interestingly, it has been suggested that the initial steps of the oxidation process could lead to an increase in antioxidant activity, whereas a decreased antioxidant capacity may be the consequence of long-lasting oxidative damage [27]. Therefore, an increase in total antioxidant capacity in glaucomatous patients may be due to a defense mechanism against oxidative damage, noting that aqueous humor presents elevated levels of the antioxidant ascorbate, up to 15-fold higher than arterial plasma [34]. On the other hand, the significant differences found between the two cohorts suggest that TAC may be a potential biomarker of oxidative stress in glaucoma. With the aim of differentiating patients with pseudoexfoliation glaucoma from healthy controls, receiver operating characteristic (ROC) curves were assessed, obtaining 22.25 μC, 18.65 μC, and 33.15 μC as the cutoff points for Q1, Q2, and Qt, respectively. Despite that larger populations of both patients and control subjects are needed, these ROC curves (Fig. S2 in ESM) show great discriminative values with 79% sensitivity and 83% specificity for Q1and 93% sensitivity and 75% specificity for Q2 and Qt.

### Elemental analysis

**Fig. 2:**
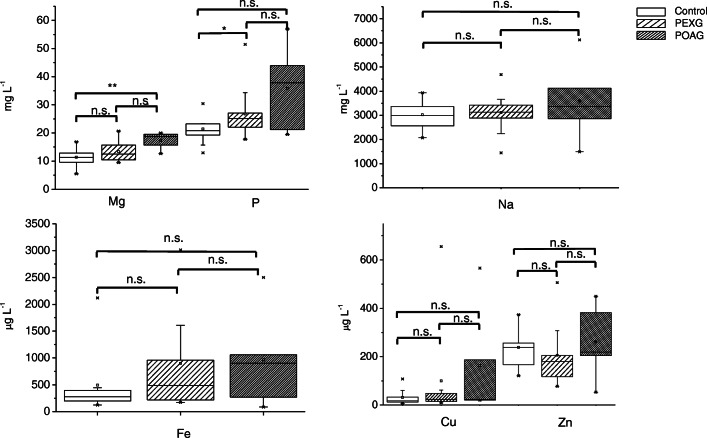
Box plots for Mg, P, and Na (mg L^−1^) and Fe, Cu, and Zn (μg L^−1^) comparing aqueous humor concentration levels of patients suffering PEXG (n = 17) and POAG (n = 5) and controls (n = 16). n.s., not significant. *p-value < 0.05; **p-value < 0.01; ***, p-value <0.001

The onset and progression of a disease could lead to alterations in the concentration of essential mineral elements due to their involvement in biological processes. In this context, possible changes in the levels of Cu, Fe, Mg, Na, P, and Zn were evaluated by comparing aqueous humor of glaucoma patients and control subjects. Table 1 compiles the results obtained, expressed as concentration range, median, first quartile, and third quartile values for PEXG, POAG, and the control group. The corresponding box plots are shown in Fig. 2. Concentrations found agree with the results reported in the literature for healthy individuals [14, 35]. The data distribution as assessed using Shapiro-Wilk’s test showed a normal distribution within the control population for almost all elements investigated, except for Cu and Fe. For the PEXG population, the concentrations of Cu, Fe, P, and Zn were not normally distributed, whereas for the POAG group, Cu was the only element for which the concentrations were not normally distributed. Significant differences were found for Mg (p = 0.003, ANOVA) and P (p = 0.038, Kruskal-Wallis). Indeed, patients with POAG showed significantly higher aqueous humor levels of Mg when compared with controls (p = 0.002, Bonferroni post hoc test), although the low number of samples for the POAG group (n = 5) should be seen as a limitation in this context. An increase in the aqueous humor levels of P was also observed in patients with PEXG in comparison with the control population (p = 0.023, Dunn post hoc test). No significant differences in the aqueous humor levels of the other trace elements studied were observed.

**Table 1:** Elemental concentration range (uncertainty: ca. 3% RSD), median, 1st quartile and 3rd quartile obtained for Mg, P, and Na (mg L^−1^) and Fe, Cu, and Zn (μg L^−1^) in PEXG, POAG, and the control group

	Group	Range	Median	1st quartile	3rd quartile
Mg	PEXG	9–21	13	10	16
	POAG	13–20	19	14	20
	Control	5–17	11	10	13
P	PEXG	18–51	25	22	29
	POAG	19–57	38	20	50
	Control	13–30	21	19	23
Na	PEXG	1447–4685	3129	2857	3504
	POAG	1489–6127	3370	2177	5127
	Control	2070–3936	3018	2565	3406
Fe	PEXG	172–3013	554	237	433
	POAG	84–2501	903	177	1404
	Control	122–2119	284	203	1781
Cu	PEXG	9–655	23	15	55
	POAG	20–566	23	20	377
	Control	6–108	18	13	53
Zn	PEXG	77–506	179	116	250
	POAG	53–449	219	129	415
	Control	121–373	243	170	294

Mg is one of the most important elements involved in the maintenance of normal metabolism and ionic balances in ocular tissues [36]. Its homeostatic alteration has been linked to pathological conditions, such as cataract, glaucoma, and diabetic retinopathy [37]. For instance, Mg deficiency has been associated with ocular disorders through several mechanisms, including oxidative stress [38]. Interestingly, it has been reported that the Mg present in aqueous humor performs a vasodilator role on the central retinal and posterior ciliary arteries [36]. On the other hand, phosphorus is crucial for numerous normal physiological functions, involving energy transfer through mitochondrial metabolism and cell signalling, among others [39]. Specifically, increased aqueous humor P levels and lower Mg levels were found in cytomegalovirus retinitis patients as compared to controls [14]. Also, higher aqueous humor P levels were reported in diabetic subjects with proliferative diabetic retinopathy [40]. Interestingly, no significant differences were found for Fe, Cu, and Zn in the present study, whereas homeostatic alterations of these elements have been reported in literature in relation to glaucoma disease. Thereby, higher aqueous humor Zn levels and lower Fe levels were reported in glaucomatous patients as compared to healthy individuals [15]. The same trend (higher values) was observed in another study for Zn and Cu, although no significant difference was found for Fe [41]. In contrast, an increased Fe level was reported in POAG patients in comparison with controls [42]. It should be stressed that a recent study did not show differences in Cu and Zn concentrations in aqueous humor from cataract patients with and without pseudoexfoliation syndrome, which is in accordance with our findings [43]. Regarding other diseases affecting the retina, statistical differences were found for Fe, Zn, and Cu in aqueous humor from AMD patients [13].

### Isotopic analysis

During the last decade, high-precision isotopic analysis of essential mineral elements has proved to provide valuable information within a clinical context. To the best of the authors’ knowledge, the search of homeostatic isotopic alterations related to ocular diseases has only been tackled in one previous publication for serum samples. In that pilot study, lower δ^65^Cu values were found in serum from AMD patients in comparison to control subjects [44]. As Cu and Zn play a key role in the visual cycle, being involved, for instance, in processes such as neurotransmission, or acting as cofactors of catalytic antioxidants [45], high-precision isotopic analysis of Cu and Zn in aqueous humor may contribute to a better understanding of ocular disorders and even help to identify potential biomarkers or new therapeutic targets. It must be noted here that, due to the low amount of sample attainable (for some samples, only 10–20 μL were available), the objective of the present work has been challenging.

**Fig. 3:**
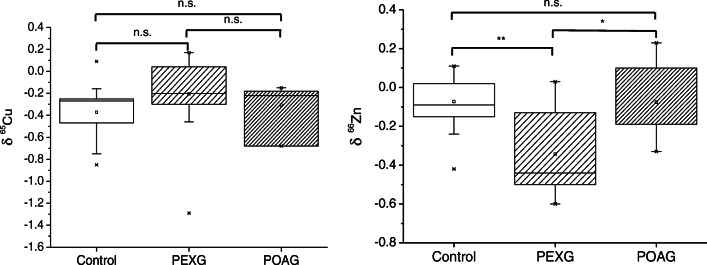
Box plots for δ^65^Cu (‰) and δ^66^Zn (‰) measured in aqueous humor of patients suffering PEXG (n = 17), of patients suffering from POAG (n = 5), and of controls (n = 16). n.s., not significant. *p-value < 0.05; **p-value < 0.01; ***p-value <0.001

Results, expressed as δ^65^Cu and δ^66^Zn, are presented in Table 2 and Fig. 3. According to our results, the Cu isotopic composition from PEXG patients is shifted towards higher values, but no significant difference was found between the three cohorts (p = 0.117, Kruskal-Wallis). In a previous study on AMD using serum, lower δ^65^Cu values were found compared to a control population. In fact, it should be mentioned that δ^65^Cu values published in serum and aqueous humor are similar [44].

**Table 2:** Gender, number of subjects, age range at the moment of sample collection, and Cu and Zn isotopic composition, expressed as δ^65^Cu (‰) and δ^66^Zn (‰) relative to NIST SRM 976 and IRMM-3702, respectively, of aqueous humor samples as obtained via MC-ICP-MS. Two isotope ratio measurement replicates were carried out whenever possible. The average internal precision obtained for δ^65^Cu and δ^66^Zn values was 0.09‰ (2se) and 0.04‰ (2se), respectively

	Gender	δ^65^Cu (range)	δ^65^Cu (mean ± SD)	δ^66^Zn (range)	δ^66^Zn (mean ± SD)	N	Age range
**PEXG**	Males	−0.46; −0.07	−0.25 ± 0.13	−0.50; 0.03	−0.28 ± 0.20	6	68–79
	Females	−1.29; 0.17	−0.18 ± 0.42	−0.60; −0.06	−0.38 ± 0.21	11	42–90
**POAG**	Males	−0.68; −0.22	−0.45 ± 0.32	−0.19; 0.10	−0.04 ± 0.21	2	70
	Females	−0.18; −0.15	−0.17 ± 0.02	−0.33; 0.23	−0.10 ± 0.29	3	69–84
**Control**	Males	−0.85; 0.09	−0.34 ± 0.11	−0.42; 0.05	−0.11 ± 0.16	7	63–75
	Females	−0.64; −0.16	−0.34 ± 0.18	−0.24; 0.07	−0.06 ± 0.11	8	34–80

However, we did find lower δ^66^Zn values in patients suffering from PEXG as compared to control subjects (*p* = 0.001, Bonferroni post hoc test). Moreover, a significant difference between the two glaucoma cohorts (PEXG vs. POAG) (p = 0.024, Bonferroni post hoc test) has also been revealed, but not when comparing POAG and controls, which highlights a specific alteration of the Zn isotopic ratio in PEXG patients. Similarly to Cu, δ^66^Zn values for the control group are in agreement with values reported for a healthy population in serum samples [21], which seems to be consistent with aqueous humor stemming in part from the plasma within the capillary network. These Zn isotope ratio alterations in the glaucomatous patients with pseudoexfoliation syndrome may be related with the antioxidant role of this element through the redox system formed with the MT proteins. It has been reported that MTs are involved in the maintenance of cellular Zn homeostasis and the defense against oxidative stress by means of neutralizing free radicals through cysteine sulphur ligands [46]. Besides, both Cu and Zn have been suggested to behave as metabolically antagonistic, acting as cofactors for the antioxidant enzyme copper-zinc SOD [13]. Although the mechanisms are not clear yet, it seems that oxidative stress and the corresponding MTs and SOD expression may be related with the isotopic Cu and Zn homeostatic alterations observed in patients diagnosed with PEXG.

During ageing, the levels of MTs decrease, while the translation of SOD seems to be upregulated, suggesting these proteins to be involved in age-related neurodegenerative pathologies, including Alzheimer’s, Parkinson’s, and retinal diseases. Oxidative stress affects the transcription and translation of these proteins [47, 48]. In the course of glaucoma onset, the SOD protein expression changes, exacerbated by ageing and oxidative stress, leading to SOD activities and levels being upregulated in the aqueous humor of glaucoma patients [32, 49]. However, there is no clear evidence showing MT changes during glaucoma development, although recently Pietrucha et al. [50] indicated that MT2 shows neuroprotective effects towards RGCs, and the levels of MTs decrease with ageing and oxidative stress [48]. These possible differences in SOD and MT levels may affect the isotopic distribution of Cu and Zn observed in our study.

The isotopic compositions of Cu and Zn in specific protein–metal complexes in the human body have not been widely studied. In a recent work, Larner et al. [51] explored the Cu isotopic composition of SOD and MTs. While the SOD copper-binding site is a nitrogen-rich histidine, the MT copper- and/or zinc-binding site is a sulphur-rich cysteine [52, 53]. Because of the stronger bonds formed, heavier isotopes will preferentially bond to amino acids with harder ligands, such as nitrogen in SOD, whereas softer ligands such as sulphur in MTs will show a preference for lighter isotopes. Therefore, the Cu isotopic composition observed in the aqueous humor of PEXG patients (enriched in the heavier isotope) may be related to SOD upregulation previously described, while the Zn isotopic composition (enriched in the lighter isotopes) may imply dysregulation of MT synthesis during glaucoma. However, the observed differences in isotopic distribution may be also related to the expression of zinc and copper transporters, and therefore, determining the expression levels of these transporters may provide further insight. In any case, the observations made here warrant further study of the isotopic composition of Cu and Zn in SOD and MTs in the context of glaucoma disease.

## Conclusions

Determination of the total antioxidant capacity in aqueous humor samples (n = 38) has been combined with their elemental and isotopic analysis with the aim of investigating potential homeostatic alterations as a consequence of glaucoma. In addition, 60 serum samples were also measured to obtain information about the total antioxidant capacity for validating the corresponding analysis results for aqueous humor. Results obtained showed higher levels of antioxidant capacity for both aqueous humor and serum in the glaucoma population, probably as a response to induced-oxidative stress damage connected to the disease. Significant differences were obtained for Qt between the control group and PEXG in aqueous humor and between controls, POAG, and PEXG in serum.

Elemental concentrations of Mg and P were established to be altered for the glaucoma cohorts as compared to the control group (also Cu, Fe, Na, and Zn were evaluated): POAG subjects showed higher levels of Mg compared with controls and PEXG patients presented higher levels of P in comparison with the control population. Isotopic compositions of Zn and Cu in aqueous humor are similar to those found in the literature for serum samples, which can be attributed to the fact that this eye fluid derives from plasma, within the capillary network of the ocular system. While no significant differences in the aqueous humor Cu isotopic composition were found when comparing the glaucoma and control groups, the isotopic composition of Zn turned out to be significantly lower for the PEXG group (δ^66^Zn values ranging between −0.60 and 0.03‰) as compared to the control group (δ^66^Zn values ranging between −0.42 and 0.11‰). Furthermore, a significant difference has also been found between the two glaucoma cohorts (PEXG vs. POAG).

Additional studies involving larger populations of POAG subjects are required to further assess the significant differences observed in Mg aqueous humor concentration and Zn isotopic composition between the two glaucoma cohorts. Moreover, it should be taken in account that, despite the meticulous selection of individuals, the results obtained could still be slightly influenced by several factors such as ethnicity, diet, geography, and lifestyle. Further studies should be carried through to further evaluate our current observations.

## Supplementary information

ESM 1 (PDF 451 kb)

## Declarations

The present study was performed according to the Declaration of Helsinki on Biomedical Research Involving Human Subjects and was approved by the Clinical Research Ethics Committee at the Principality of Asturias (Oviedo, Spain).
