# Supplementary material for: Homeostatic alterations related to total antioxidant capacity, elemental concentrations and isotopic compositions in aqueous humor of glaucoma patients
Source: Anal Bioanal Chem. 2021 Jun 25;414(1):515–24. doi: 10.1007/s00216-021-03467-5 (PMC8748375; doi:10.1007/s00216-021-03467-5)

Electronic Supplementary Material

Homeostatic alterations related to total antioxidant capacity, elemental concentrations and isotopic compositions in aqueous humor of glaucoma patients

Marta Aranaz[1], Marta Costas-Rodríguez[2*], Lara Lobo[1*], Montserrat García[3,4], Héctor González-Iglesias[3,4], Rosario Pereiro[1,4] and Frank Vanhaecke[2]

[1]Department of Physical and Analytical Chemistry, University of Oviedo, Avda. Julián Clavería 8, 33006 Oviedo, Spain

[2]Department of Chemistry, Atomic & Mass Spectrometry – A&MS research unit, Ghent University, Campus Sterre, Krijgslaan 281 - S12, 9000 Ghent, Belgium

[3]Instituto Oftalmológico Fernández-Vega, Avda. Fernández-Vega 34, 33012 Oviedo, Spain

[4]Instituto Universitario Fernández-Vega, Fundación de Investigación Oftalmológica, Universidad de Oviedo, Spain

*Corresponding author:

lobolara@uniovi.es, Phone: +34985105366

Marta.CostasRodriguez@ugent.be, Phone: +32(0)926246595

Table S1. Demographic features for the patients with pseudoexfoliation glaucoma (PEXG) or primary open angle glaucoma (POAG) and the control subjects

	PEXG (n=17)	POAG (n=5)	Control (n=16)	[1]p-value	[2]p-value	[3]p-value
Gender (male/female)	6/11	2/3	7/8	0.770	1.000	1.000
Age (mean years ± SD)	72±11	75±7	66±11	0.508	0.049	0.804
Age range	42-90	69-84	34-80	-	-	-
Diabetes	1	0	0	1.000	-	1.000
Myopia	0	1	2	0.212	1.000	0.227
Arterial hypertension	2	0	1	1.000	1.000	1.000
Dyslipidemia	3	0	3	1.000	0.540	0.540
Rheumatic/inflammatory diseases	1	0	0	1.000	-	1.000
Osteoporosis	1	0	0	1.000	-	1.000
Hepatic cirrhosis	1	0	0	1.000	-	1.000
Hernia	2	0	0	0.485	-	1.000

n, number of subjects; SD, standard deviation.[1](Control vs PEXG), [2](Control vs POAG), [3](PEXG vs POAG)

Table S2. Demographic characteristics of patients with pseudoexfoliation glaucoma (PEXG), primary open angle glaucoma (POAG) and control subjects recruited for the antioxidant capacity analysis of serum.

	PEXG (n=20)	POAG (n=20)	Control (n=20)	[1]p-value	[2]p-value	[3]p-value
Gender (male/female)	11/9	12/8	10/10	0.752	0.751	0.749
Age (mean years ± SD)	75 ± 7	72±8	72±8	0.139	0.735	0.336
Age range	61-85	53-85	62-92	-	-	-
Diabetes	1	2	3	1.000	0.633	0.598
Myopia	3	6	2	0.633	0.236	0.449
Arterial hypertension	10	8	9	0.752	0.750	0.751
Dyslipidemia	9	7	8	1.000	0.744	0.747
Rheumatic/inflammatory diseases	2	1	1	0.548	1.000	0.548
Osteoporosis	1	0	0	0.311	-	0.311
Hepatic cirrhosis	1	0	0	0.311	-	0.311
Hernia	0	0	0	-	-	-

n, number of subjects; SD, standard deviation.[1](Control vs PEXG), [2](Control vs POAG), [3](PEXG vs POAG)

Figure S1. Antioxidant capacity levels in serum samples (n=20 controls; n=20 PEXG; N=20 POAG) obtained using the Antioxidant Capacity Assay Kit (panel A, trolox levels in nmol) and the e-BQC device (Qt in panel B, Q1 in panel C and Q2 in panel D). n.s., not significant; *, p-value < 0.05; **, p-value < 0.01; ***, p-value < 0.001.

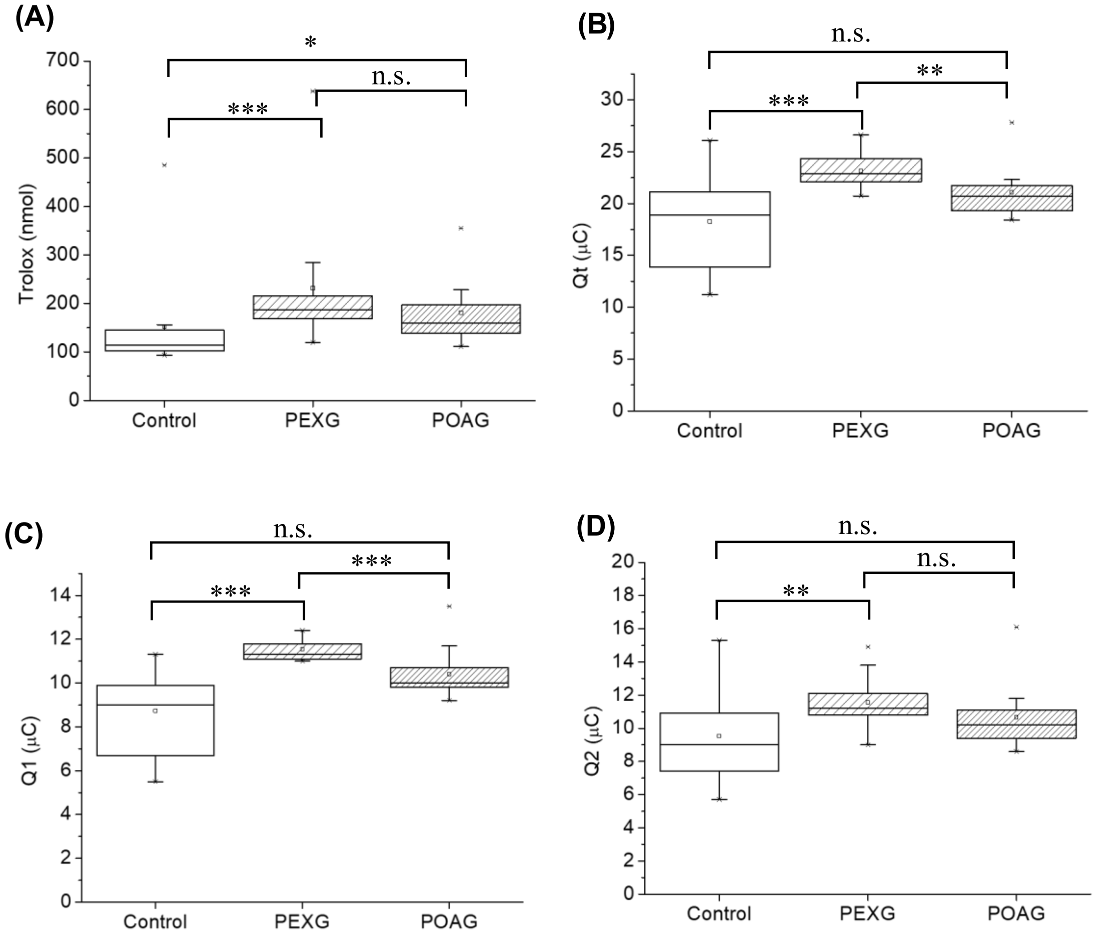

Fig. S2. ROC curves for Q1, Q2 and Qt, respectively (AUC=0.860; AUC=0.875; AUC=0.869). Although larger populations of both patients and control subjects are needed, these ROC curves show great discriminative values with 81% sensitivity and 83% specifity for Q1; 93% sensitivity and 75% specificity for Q2 and Qt.

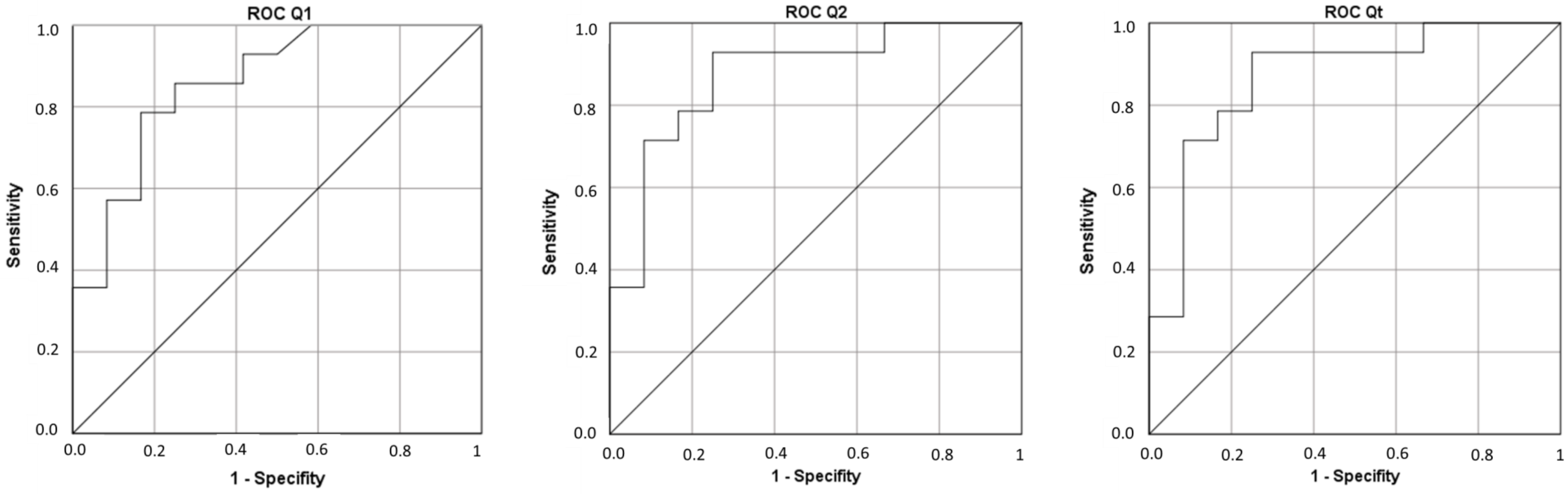

Supplement: Supplementary file 1 — (PDF 451 kb) [file 216_2021_3467_MOESM1_ESM.pdf]
